# Photonic Nanochains for Continuous Glucose Monitoring in Physiological Environment

**DOI:** 10.3390/nano14110964

**Published:** 2024-06-01

**Authors:** Gongpu Shi, Luying Si, Jinyang Cai, Hao Jiang, Yun Liu, Wei Luo, Huiru Ma, Jianguo Guan

**Affiliations:** 1 State Key Laboratory of Advanced Technology for Materials Synthesis and Processing, International School of Materials Science and Engineering, Wuhan University of Technology, Wuhan 430070, China; 270578@whut.edu.cn (G.S.); siluying@whut.edu.cn (L.S.); jh1998@whut.edu.cn (H.J.); liuyun650403@whut.edu.cn (Y.L.); guanjg@whut.edu.cn (J.G.); 2 School of Materials Science and Engineering, Wuhan University of Technology, Wuhan 430070, China; caijinyang@whut.edu.cn; 3 School of Chemistry, Chemical Engineering and Life Science, Wuhan University of Technology, Wuhan 430070, China; 4 Wuhan Institute of Photochemistry and Technology, 7 North Bingang Road, Wuhan 430083, China

**Keywords:** glucose monitor, responsive photonic nanochains, colorimetric sensor, physiological environment

## Abstract

Diabetes is a common disease that seriously endangers human health. Continuous glucose monitoring (CGM) is important for the prevention and treatment of diabetes. Glucose-sensing photonic nanochains (PNCs) have the advantages of naked-eye colorimetric readouts, short response time and noninvasive detection of diabetes, showing immense potential in CGM systems. However, the developed PNCs cannot disperse in physiological environment at the pH of 7.4 because of their poor hydrophilicity. In this study, we report a new kind of PNCs that can continuously and reversibly detect the concentration of glucose (C_g_) in physiological environment at the pH of 7.4. Polyacrylic acid (PAA) added to the preparation of PNCs forms hydrogen bonds with polyvinylpyrrolidone (PVP) in Fe_3_O_4_@PVP colloidal nanoparticles and the hydrophilic monomer *N*-2-hydroxyethyl acrylamide (HEAAm), which increases the content of PHEAAm in the polymer shell of prepared PNCs. Moreover, 4-(2-acrylamidoethylcarbamoyl)-3-fluorophenylboronic acid (AFPBA), with a relatively low pKa value, is used as the glucose-sensing monomer to further improve the hydrophilicity and glucose-sensing performances of PNCs. The obtained Fe_3_O_4_@(PVP-PAA)@poly(AFPBA-co-HEAAm) PNCs disperse in artificial serum and change color from yellow-green to red when C_g_ increases from 3.9 mM to 11.4 mM, showing application potential for straightforward CGM.

## 1. Introduction

Diabetes is a metabolic disease characterized by hyperglycemia, and its main harm lies in various complications caused by long-term hyperglycemia [1]. Due to the increasing incidence of diabetes worldwide [2,3], continuous glucose monitoring (CGM) in the human body is becoming more and more important [4]. At present, the mainstream glucose sensors are divided into electrochemical sensors and optical sensors, among which electrochemical glucose sensors have attracted widespread attention for their low cost, rapid response and ease of use [5]. Electrochemical enzymatic reactions based on glucose and glucose oxidase or glucose dehydrogenase have been commercialized for blood glucose detection [6,7,8]. But these enzymes are easy to lose activity at temperatures above 40 °C and in acidic or alkaline environments [9,10], and immobilizing enzymes onto the surface of sensors is also a complex process [11]. Although many enzyme-free nanomaterials have been developed for the direct electrochemical oxidation of glucose [12,13,14], most of them cannot work under physiological pH and have poor selectivity [15]. These drawbacks greatly limit the long-term use of electrochemical glucose sensors in CGM systems. Optical glucose-sensing technologies, including fluorescence and surface plasmon resonance [16,17,18], detect the concentration of glucose (C_g_) based on changes in the properties of photons. They have the advantages of both sensitivity and versatility, enabling fast blood glucose monitoring [19,20]. However, fluorescent dyes suffer from the disadvantages of light bleaching and chemical instability, and most optical glucose sensors are large in size, expensive, have poor signal-to-noise ratio (SNR), and require frequent invasive calibration [21,22].

Among various sensing mechanisms and architectures, glucose-sensing photonic crystals (PCs) bearing phenylboronic acid (PBA) have been demonstrated to be promising glucose sensors [23,24]. The structural color of PCs can continuously and reversibly change with C_g_ in the entire visible spectrum, which corresponds to the change of the hydrogel volume caused by the interaction between PBA and glucose [23,25]. This colorimetric detection for determining C_g_ is visual, simple, and practical. Additionally, glucose-sensing PCs require no additional energy consumption, have a high SNR and are not affected by light bleaching or chemical instability [26,27]. Due to the low cost of self-assembly methods, the combination of colloidal crystal arrays (CCAs) and PBA-modified hydrogels for preparing glucose-sensing PCs has been extensively studied [25,28]. The preparation process of PBA-modified 3-D CCAs and inverse opal PCs is time-consuming, typically taking several weeks to reach a good structure of PCs [29]. In addition, 2D monolayer CCAs can be continuously generated at the air/water interface within a few minutes [30,31], but it is difficult to maintain highly ordered single-layer CCAs. At the same time, the thickness of the above glucose-sensing hydrogels with PCs is at the micrometer level, leading to a relatively long diffusion distance for glucose molecules; therefore, several minutes or hours are required to reach the equilibrium of the response, which makes it difficult to achieve CGM [32]. In contrast, 1D glucose-sensing photonic nanochains (PNCs) can reach the equilibrium within seconds, and the structural color of the PNCs can continuously and reversibly change with C_g_, providing the potential to realize CGM [33]. PNCs have also been applied to detect C_g_ in microenvironments due to their characteristics of miniaturization [34].

However, the existing glucose-sensing PNCs have poor hydrophilicity and cannot disperse in physiological environment at the pH of 7.4 because of the lack of hydrophilic *N*-2-hydroxyethyl acrylamide (HEAAm) in the polymer shell and the use of 3-acrylamido phenylboronic acid (AAPBA) with a relatively high pKa value, which severely affects their practical applications. It has been proved that polyacrylic acid (PAA) can form strong hydrogen bonds with the carbonyl groups of polyvinylpyrrolidone (PVP) and the amide groups of *N*-isopropyl acrylamide [35,36]. Herein, we hypothesized that PAA can form strong hydrogen bonds with the amide groups of HEAAm too. To improve the hydrophilicity of the hydrogel in the PNCs, we added a trace of PAA during the preparation of glucose-sensing PNCs. PAA formed hydrogen bonds with PVP and HEAAm, leading to the enrichment of HEAAm within the PVP brush shells of Fe_3_O_4_@PVP particles, which increased the content of PHEAAm in the prepared PNCs. In addition, we selected the glucose-sensing monomer 4-(2-acrylamidoethylcarbamoyl)-3-fluorophenylboronic acid (AFPBA) with a pKa value of 7.2 instead of AAPBA with a pKa value of 8.2 to further improve the hydrophilicity and glucose-sensing performances of the PNCs [37,38,39]. The obtained Fe_3_O_4_@(PVP-PAA)@poly(AFPBA-co-HEAAm) PNCs can disperse in phosphate buffered solution at the pH of 7.4, undergo a wide wavelength shift of 120 nm when C_g_ changes from 0 to 20 mM and reach equilibrium in seconds. The PNCs can also work in artificial serum and change color from yellow-green to red. This work provides a possibility for glucose-sensing PNCs to carry out CGM in physiological environment and microenvironments at the pH of 7.4.

## 2. Materials and Methods

### 2.1. Materials

The following materials were purchased from Aladdin Reagent (Shanghai, China) Co., Ltd.: 3-acrylamido phenylboronic acid (AAPBA), *N*-(2-hydroxyethyl) acrylamide (HEAAm), 2-hydroxy-2-methylpropiophenone (HMPP), *N*,*N*’-methylenebisacrylamide (BIS), disodium hydrogen phosphate dodecahydrate (Na_2_HPO_4_·12H_2_O), disodium phosphate dihydrate (NaH_2_PO_4_·2H_2_O) and a poly(acrylic acid) aqueous solution (PAA, Mw~3 kDa, 50 wt% in H_2_O). D(+)-glucose, D-(+)-galactose, D-fructose, sucrose and dimethyl sulfoxide (DMSO) were provided from Sinopharm Chemical Reagent Co., Ltd. (Shanghai, China) 4-(2-acrylamidoethylcarbamoyl)-3-fluorophenylboronic acid (AFPBA) was obtained from Bide Pharmatech Co., Ltd. (Shanghai, China) Artificial serum was purchased from Braveds Biotechnology (Shenzhen, China) Co., Ltd. All chemicals were used as received. Deionized water was produced in a Milli-Q system. Superparamagnetic Fe_3_O_4_@PVP colloidal nanoparticles (CNCs) with an average particle size of 140 nm (Figure S1) were prepared by a modified polyol process according to our previous report [40]. Phosphate-buffered (PBS) solutions at different pH values and 150 mM of ionic strength were prepared according to previous research [41].

### 2.2. Preparation of the Prepolymerization Solution

A stock solution of Fe_3_O_4_@PVP CNCs was obtained by centrifuging 1 mL of ethanol solution containing Fe_3_O_4_@PVP CNCs at the concentration of 10 g L^−1^ and then redispersing it into DMSO (0.39 mL) under sonication. The aqueous solution of 50 mg L^−1^ PAA was obtained by dispersing 25 mg of PAA aqueous solution (50 wt% in H_2_O) in 250 mL of deionized water. Different concentrations of the PAA aqueous solution were obtained by adjusting the amount of PAA added. The stock solutions of monomers or photoinitiator were produced by dissolving 0.14 g of AFPBA, 0.49 g of HEAAm, 0.015 g of BIS and 0.033 g of HMPP in 545, 181, 500 and 500 μL of DMSO.

### 2.3. Preparation of Fe_3_O_4_@(PVP-PAA)@poly(AFPBA-co-HEAAm) PNCs

In a typical synthesis of PNCs, 16.0 μL of the Fe_3_O_4_@PVP CNCs stock solution was mixed with 27.5 μL of the AFPBA stock solution, 15.1 μL of the HEAAm stock solution, 13.1 μL of the BIS stock solution, 10.2 μL of the HMPP stock solution, 29.7 μL of DMSO and 900 μL of the 50 mg L^−1^ PAA aqueous solution in a 10 mL glass beaker to form a prepolymer solution by sonication. After that, the glass beaker was placed above the center of a 10 × 10 × 2 cm rectangular NdFeB permanent magnet providing a magnetic field (*H*) strength of 500 Gs for 1 min, followed by UV light irradiation for 5 min. Then, 2 mL of DMSO was added for dilution, and the final Fe_3_O_4_@(PVP-PAA)@poly(AFPBA-co-HEAAm) PNCs were magnetically separated from the solution. Different products could be obtained by changing the concentration of HEAAm in the prepolymer solution or the concentration of the PAA aqueous solution.

### 2.4. Characterizations

All digital photos in this paper were taken by using iPhone 14 pro. The scanning electron microscope (SEM) images were acquired by a field-emission scanning electron microscope (FE-SEM, Hitachi S-4800, 10 kV, Hitachi, Tokyo, Japan). The transmission electron microscope (TEM) images were taken on a high-resolution transmission electron microscope (HRTEM, JEOL JEM-2100F, 200 kV, JEOL Ltd., Tokyo, Japan). The Fourier transform infrared (FTIR) spectra in the range of 400–4000 cm^−1^ with a resolution of 4 cm^−1^ were obtained by a 60-SXBFTIR spectrometer (Thermo Fisher Scientific, Waltham, MA, USA). The thermal analyses were performed by a NETZSCH-STA449C/G instrument (NETZSCH, Selb, Germany) under air from room temperature to 1000 °C, at a heating rate of 5 °C min^−1^. The inductively coupled plasma (ICP) data were obtained on an inductively coupled plasma optical emission spectrometer (SPECTRO BLUE SOP, Kleve, Germany). The dark-field optical microscope images were captured by using an optical microscope (Zeiss Axio Observer 5M, Oberkochen, Germany).

### 2.5. Optical Properties

The PNCs were dispersed in PBS buffers at different pH, recording reflection spectra at different concentrations of glucose by using a fiber optic spectrometer (Ocean Optics, Orlando, FL, USA; USB 2000+) under a 300 Gs magnetic field.

### 2.6. Calculation of the Content of Each Component in the Fe_3_O_4_@(PVP-PAA)@poly(AFPBA-co-HEAAm) PNCs

The weight percentage of Fe_3_O_4_ and polymer in the PNCs was determined by thermogravimetric analysis (TGA). Since the weight ratio of Fe and B was obtained by ICP, we calculated the weight percentage of AFPBA through the weight percentage of Fe_3_O_4_ in the PNCs. The weight ratio between PVP and Fe_3_O_4_ was determined through TGA of the Fe_3_O_4_@PVP CNCs. Thus, the weight percentage of PVP was calculated from the weight percentage of Fe_3_O_4_ in the PNCs too. Because the amount of PAA in the PNCs was negligible, the weight percentage of HEAAm was estimated by subtracting the weight percentage of Fe_3_O_4_, AFPBA and PVP from the total weight.

### 2.7. Calculation of Hydrogen Bond Energies

In order to calculate the hydrogen bond energies between HEAAm, PVP and PAA, we used the atoms-in-molecules (AIM) theory to study electron density (ρ_BCP_) at the bond critical point of the hydrogen bonds [42]. At first, the chemical structures were optimized using the def2svp basis set and the M06-2X functional in Gaussian09 (Revision D.01) [43,44,45]. Frequency calculations were performed to check that all the geometries corresponded to energy minima. Then, the single point energy calculation of the optimized chemical structure was performed to obtain the Gaussian output fch-file. The Gaussian output fch-file was used as the input in the Multiwfn (Version 3.7) program to obtain ρ_BCP_ through AIM analysis [46]. At last, ρ_BCP_ was substituted into the prediction equation:

E_HB = −223.08 × ρ_BCP_ + 0.7423 (1)

(where the hydrogen bond energy E_HB is in kcal/mol, and ρ_BCP_ is in a.u.) to estimate the hydrogen bond energy [42].

## 3. Results and Discussion

### 3.1. Formation and Response Mechanism of Fe_3_O_4_@(PVP-PAA)@poly(AFPBA-co-HEAAm) PNCs after the Addition of PAA and AFPBA 

**Figure 1:**
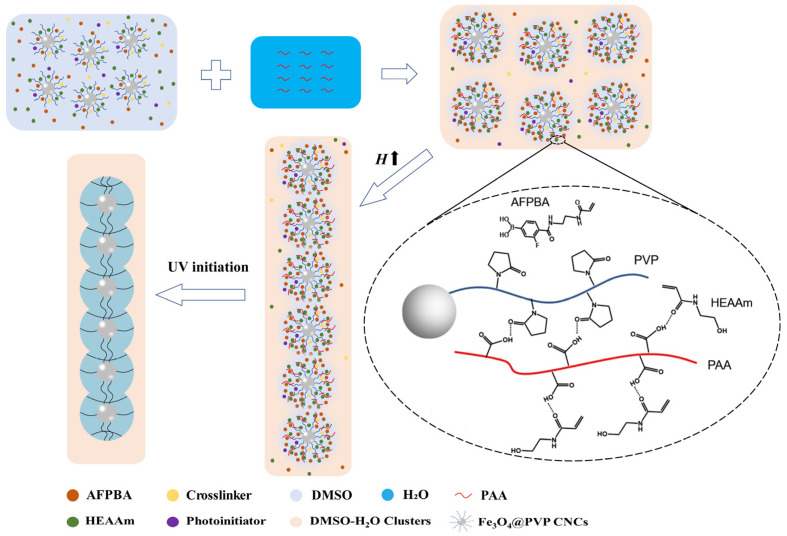
Schematic illustration of the formation of PNCs after the addition of PAA.

Figure 1 shows the mechanism of the formation of PNCs after PAA addition. At first, all monomers, crosslinker, and photoinitiator were uniformly dispersed in DMSO solvent. Upon adding the PAA aqueous solution, most DMSO molecules formed DMSO-H_2_O clusters with H_2_O through hydrogen bonding interactions. A small volume of free DMSO remained around the Fe_3_O_4_@PVP CNCs [33]. AFPBA accumulated around the Fe_3_O_4_@PVP CNCs along with free DMSO. At the same time, PAA formed hydrogen bonds with the carbonyl groups of PVP and the amide groups of HEAAm, leading to the enrichment of hydrophilic HEAAm within the PVP brush shells. Then, under the influence of a magnetic field, the Fe_3_O_4_@PVP CNCs assembled into a 1D chain-like structure, followed by in situ polymerization with UV light to form Fe_3_O_4_@(PVP-PAA)@poly(AFPBA-co-HEAAm) PNCs.

**Figure 2:**
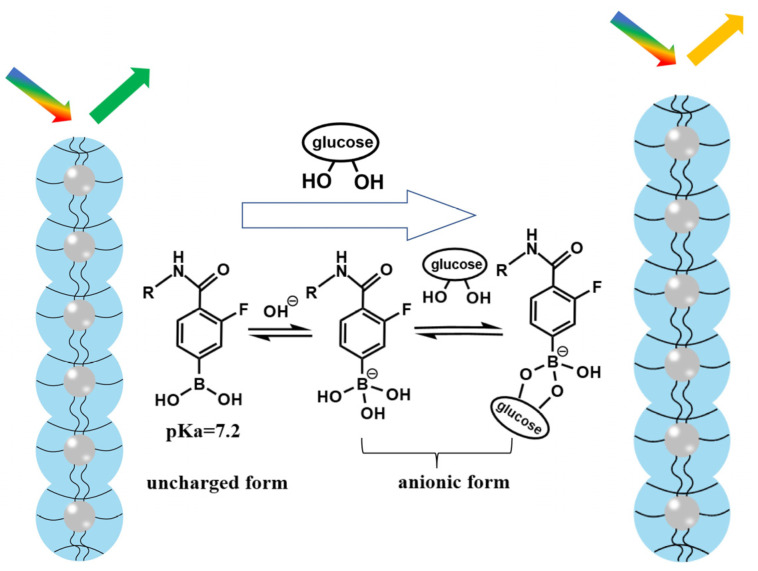
Schematic illustration of the glucose response induced by AFPBA.

As shown in Figure 2, AFPBA was present in hydrophobic uncharged form and hydrophilic borate anions on the PNCs. The pKa value of AFPBA is 7.2, which is much lower than that (8.2) of AAPBA. Therefore, compared with AAPBA, AFPBA provides more hydrophilic borate anions in an environment at the pH of 7.4 (Table S1). The borate anions can easily combine with glucose to form stable complexes, promoting further AFPBA dissociation and the production of more hydrophilic borate anions, which improves the hydrophilicity of the polymer on PNCs [47,48,49]. The increase in hydrophilicity will make PNCs absorb water and swell, resulting in a red shift in wavelength (Figure 2). So, choosing AFPBA as the glucose-sensing monomer instead of AAPBA will provide PNCs with better hydrophilicity and glucose responsiveness.

### 3.2. Characterization and Glucose-Sensing Performances of Typical Fe_3_O_4_@(PVP-PAA)@poly(AFPBA-co-HEAAm) PNCs

**Figure 3:**
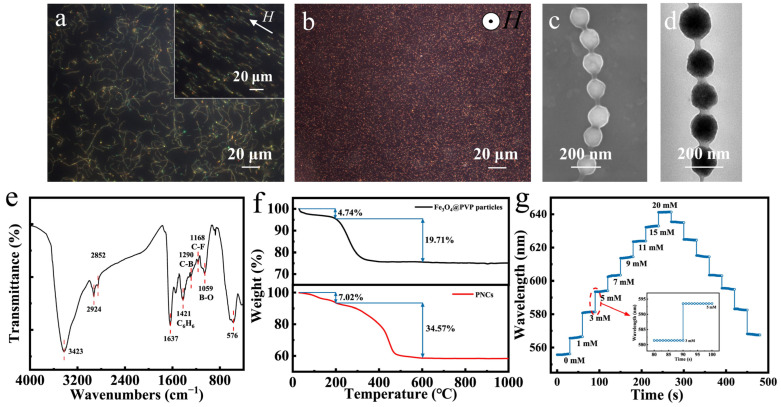
Characterization and glucose-sensing performances of typical Fe_3_O_4_@(PVP-PAA)@poly(AFPBA-co-HEAAm) PNCs. (**a**,**b**) Dark-field optical microscope images of PNCs under different magnetic fields; (**c**) SEM image of PNCs; (**d**) TEM image of PNCs; (**e**) FTIR spectrum; (**f**) TG curves of Fe_3_O_4_@PVP CNCs and PNCs; (**g**) glucose-sensing performances of typical PNCs in PBS buffer at the pH of 7.4.

Figure 3a,b present dark-field optical microscope images of typical Fe_3_O_4_@(PVP-PAA)@poly(AFPBA-co-HEAAm) glucose-sensing PNCs which were fabricated in a 50 mg L^−1^ PAA aqueous solution with a feed molar ratio of HEAAm to AFPBA of 4.0. Almost all PNCs exhibited a random curved state without a magnetic field (Figure 3a). After applying a horizontal magnetic field, the PNCs aligned to the direction of the applied magnetic field and became straight, with an average length of 30–35 μm (Figure 3a). These phenomena indicated that the prepared PNCs possessed both magnetic and flexible properties. When the magnetic field changed to a vertical direction, the PNCs aligned perpendicular to the plane along the direction of the magnetic field, resulting in the appearance of many bright red dots (Figure 3b). Figure 3c shows the SEM image of the PNCs. It can be observed that the PNCs had a morphology similar to that of a pod, exhibiting a 1D ordered structure. The distance between the particles was also mostly uniform, indicating that the colors was generated due to the periodic structure of the PCs. From the TEM image in Figure 3d, it can be seen that the PNCs were composed of Fe_3_O_4_ CNCs as the core combined with an organic layer with lower contrast. All CNCs were covered and connected by an organic layer of about 15–20 nm. Then, we used FTIR to investigate the chemical composition of the Fe_3_O_4_@(PVP-PAA)@poly(AFPBA-co-HEAAm) PNCs (Figure 3e). The absorption peaks at 3423 cm^−1^ and 1637 cm^−1^ were due to the stretching vibrations of N-H and C=O in the amide group of HEAAm [50]. The absorption peaks at 1421 cm^−1^, 1290 cm^−1^, 1168 cm^−1^ and 1059 cm^−1^ correspond to the stretching vibrations of the benzene ring skeleton and of the C-B, C-F and B-O bonds of AFPBA, respectively [39]. The strong absorption peak at 576 cm^−1^ was attributed to the stretching vibration of the Fe-O bonds of Fe_3_O_4_ [40]. Therefore, it can be inferred that the polymer layer of the PNCs was mainly composed of PVP and poly(AFPBA-co-HEAAm).

The thermogravimetric (TG) curves of Fe_3_O_4_@PVP CNCs and typical Fe_3_O_4_@(PVP-PAA)@poly(AFPBA-co-HEAAm) PNCs are shown in Figure 3f. These samples were heated from room temperature to 1000 °C at a rate of 5 °C min^−1^ under air. In the upper part of Figure 3f, the TG curve of Fe_3_O_4_@PVP CNCs shows two weight loss plateaus as the temperature increased. The first weight loss plateau from 0 to 200 °C was mainly attributed to the evaporation of free and structural water inside the CNCs, indicating that water accounted for 4.74% of the total weight. The second weight loss plateau from 200 to 600 °C was primarily due to the decomposition of the polymer inside the CNCs, revealing that the polymer inside the CNCs accounted for 19.71% of the total weight. Therefore, based on the TG curve of the CNCs, we calculated that the Fe_3_O_4_@PVP CNCs were composed of 20.7 wt% PVP and 79.3 wt% Fe_3_O_4_. In the lower part of Figure 3f, the TG curve of the Fe_3_O_4_@(PVP-PAA)@poly(AFPBA-co-HEAAm) PNCs also exhibits two weight loss plateaus as the temperature increased. The first weight loss plateau corresponds to the loss of free and structural water within the nanochains, accounting for 7.02% of the total weight. The second weight loss plateau was attributed to the decomposition of the polymer, representing 34.57% of the total weight. The polymer within the PNCs included PVP, poly(AFPBA-co-HEAAm) and trace amounts of PAA. Based on the TG curve of the PNCs, we calculated that the Fe_3_O_4_@(PVP-PAA)@poly(AFPBA-co-HEAAm) PNCs were primarily composed of 62.82 wt% Fe_3_O_4_ and 37.18 wt% polymer. Figure 3g depicts the glucose-sensing performances of typical Fe_3_O_4_@(PVP-PAA)@poly(AFPBA-co-HEAAm) PNCs in PBS buffer at the pH of 7.4. As C_g_ increased from 0 mM to 20 mM, the wavelength shifted from 556.3 nm to 641.4 nm, continuously and reversibly. The total wavelength shift reached 85.1 nm, covering the visible spectrum from green to red. The inset in Figure 3g demonstrates the time required for the PNCs to reach equilibrium. It can be seen that as C_g_ changed, the diffraction peak immediately shifted and remained relatively constant for various seconds, indicating that equilibrium was reached. Additionally, during cyclic changes in C_g_, the diffraction peak was consistent at the same C_g_ point, demonstrating the excellent reversibility of the glucose-sensing PNCs. To investigate the selectivity of the glucose-sensing PNCs to other sugar molecules, we used the PNCs to detect structural analogues of glucose, including galactose, fructose and sucrose. The PNCs exhibited higher sensitivity for galactose and fructose compared to glucose, with wavelength shifts of 126 and 168 nm (Figure S2). This is because fructose and galactose have higher affinity for phenylboronic acid (PBA) than for glucose [51]. Fortunately, the concentrations of galactose and fructose in human fluids are very low, almost one order of magnitude smaller than that of glucose [52]. So, fructose and galactose will hardly cause a signal drift when using PNCs for the detection of glucose in human fluids.

### 3.3. Explanation and Verification of the Mechanism of PAA-Induced PNCs Formation

**Figure 4:**
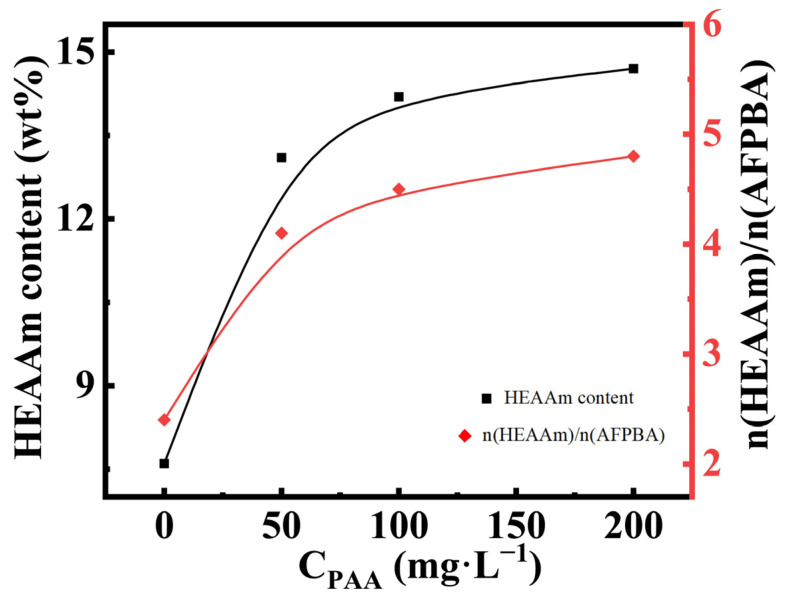
The content of HEAAm and the molar ratio of HEAAm to AFPBA in the PNCs prepared under different C_PAA_ with a feed molar ratio of HEAAm to AFPBA of 4.0.

The typical Fe_3_O_4_@(PVP-PAA)@poly(AFPBA-co-HEAAm) PNCs mentioned earlier were prepared in a 50 mg L^−1^ PAA aqueous solution with a feed molar ratio of HEAAm to AFPBA of 4.0. In order to verify the mechanism of action of PAA proposed above, we used TG analysis (Figure S3) and ICP analysis (Table S2) of the PNCs prepared at different concentrations of the PAA (C_PAA_) aqueous solutions. Based on these data, we calculated the specific content of each component in the PNCs through the method described in Section 2.7 (Table S3). Figure 4 shows the changes in the weight percentage of HEAAm and the molar ratio of HEAAm to AFPBA in the PNCs when C_PAA_ increased during the preparation of the PNCs. When preparing PNCs without the addition of PAA, the molar ratio of HEAAm to AFPBA was 2.4, much lower than the feed molar ratio of 4.0. This was mainly because HEAAm tended to disperse evenly in the pre-polymer solution, while AAPBA tended to concentrate around the Fe_3_O_4_@PVP CNCs [33]. As C_PAA_ increased during the preparation of the PNCs, it could be observed that the content of HEAAm in the PNCs significantly increased. This indicated that the addition of PAA allowed HEAAm to more effectively concentrate around the Fe_3_O_4_@PVP CNCs, resulting in the prepared PNCs containing more PHEAAm. The final molar ratio of HEAAm to AFPBA was slightly higher than 4.0. This could be due to the presence of a small amount of PAA in the prepared PNCs, which was included in the HEAAm content during the calculation process.

**Figure 5:**
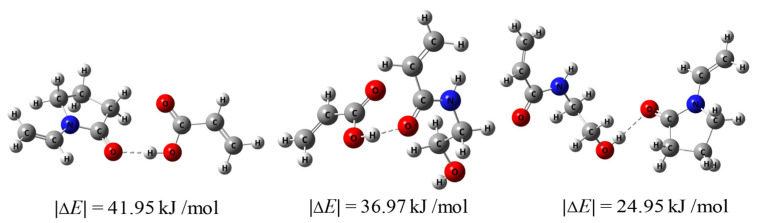
The hydrogen bond energies between different compounds calculated using Gaussian09 (Revision D.01) and Multiwfn (Version 3.7).

Figure 5 shows the calculated hydrogen bond energies. It can be observed that the hydrogen bond energy between HEAAm and PVP alone was 24.95 kJ/mol, which was similar to the hydrogen bond energy between HEAAm, H_2_O and DMSO (Figure S4). This proved that HEAAm tended to be uniformly dispersed in the solution without the addition of PAA. The hydrogen bond energy between PAA and PVP was 41.95 kJ/mol, and the hydrogen bond energy between PAA and HEAAm was 36.97 kJ/mol, which indicated that PAA established strong hydrogen bonding interactions with PVP and HEAAm. Therefore, PAA can act as a bridge and enable the uniformly dispersed HEAAm in the solution to concentrate around the PVP brush shells of the Fe_3_O_4_@PVP CNCs through hydrogen bonding interactions.

**Figure 6:**
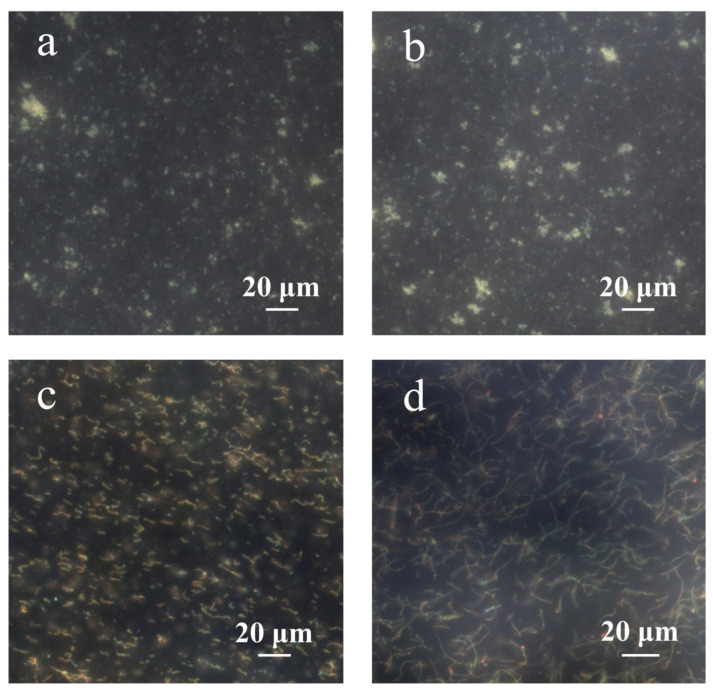
The dark-field microscopic images of PNCs prepared under different conditions. (**a**) AFPBA and PAA were not added, but HEAAm and the crosslinker BIS were retained; (**b**) only the PAA aqueous solution was added; (**c**) AFPBA and HEAAm were not added, but PAA and BIS were retained; (**d**) AFPBA and BIS were not added, but PAA and HEAAm were retained.

Figure 6 depicts the dark-field microscopic images of PNCs prepared under different conditions. According to a previous study, the chain structure cannot be obtained without the addition of hydrophobic AAPBA in the preparation of glucose-sensing PNCs [33]. As shown in Figure 6a, during the preparation of PNCs, if AFPBA and PAA were not added, but HEAAm and the crosslinker BIS were retained, no chain structure formed. This was because neither HEAAm nor BIS could form strong hydrogen bonds with PVP and concentrate around the Fe_3_O_4_@PVP CNCs; therefore, the concentration of monomers around the CNCs was the same as that in the solution, and the polymer could not preferentially form around the Fe_3_O_4_@PVP CNCs to produce a chain structure. Figure 6b shows that if only the PAA aqueous solution was added in the preparation of PNCs, that is, AFPBA, HEAAm and BIS were not added, no chain structure was observed, because PAA is not a monomer and could not be directly used for the synthesis of the polymer. Figure 6c shows that many short-chain structures formed during the preparation of PNCs when removing AFPBA and HEAAm while retaining BIS and PAA. Interestingly, BIS also contains amide groups, indicating that PAA and BIS exhibit similar hydrogen bonding interactions, as described earlier. This led to the enrichment of BIS around the CNCs, resulting in the formation of PNCs. Due to the low content of the crosslinker BIS, the strength of the chains was insufficient, resulting in short PNCs. In Figure 6d, when AFPBA and the crosslinker BIS were removed during the preparation process, and HEAAm and PAA were retained, a significant number of long chains formed. This further confirmed the successful enrichment of HEAAm around the Fe_3_O_4_@PVP CNCs, providing experimental evidence that PAA can form strong hydrogen bonds with PVP and HEAAm. Additionally, this strong hydrogen bond interactions exist in other compounds containing amide groups, such as BIS.

### 3.4. Effects of Preparation Conditions and pH on the Dispersibility and Glucose-Sensing Performances of the Fe_3_O_4_@(PVP-PAA)@poly(AFPBA-co-HEAAm) PNCs

**Figure 7:**
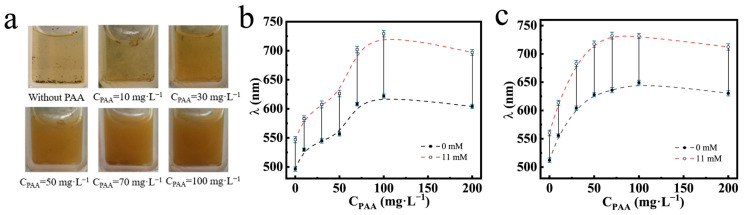
The Fe_3_O_4_@(PVP-PAA)@poly(AFPBA-co-HEAAm) PNCs prepared at different C_PAA_. (**a**) Dispersion of the PNCs without magnetic field and glucose in PBS buffer at the pH of 7.4; change in the diffraction wavelength of the PNCs when Cg increased from 0 mM to 11 mM in PBS buffer at the pH of 7.4; (**b**) PNCs prepared in the feed molar ratio of HEAAm to AFPBA of 4.0; (**c**) PNCs prepared in the feed molar ratio of HEAAm to AFPBA of 4.5.

The dispersibility and glucose-sensing performances of the PNCs prepared at different C_PAA_ and feed molar ratios are shown in Figure 7. The intrinsic color of the PNCs dispersed in solution was brown. In Figure 7a, we can see that as C_PAA_ increased during the preparation of the PNCs, the PNCs dispersed in PBS buffer at the pH of 7.4, and the color of the solution changed from transparent to brown, indicating that the dispersion progressively increased. Figure 7b shows the glucose-sensing performances of the PNCs prepared at different C_PAA_ at the feed molar ratio of HEAAm to AFPBA of 4.0. It can be observed that as C_PAA_ increased, the initial diffraction peak position and the wavelength shift of the PNCs gradually increased and then stabilized. This was because that the addition of PAA enhanced the hydrophilicity and the content of the PHEAAm gel in the PNCs, causing water absorption and the expansion of the hydrogel layer, resulting in the red shift of the initial diffraction peak. Additionally, the improved dispersibility of the PNCs and the better swelling property of the hydrogel layer led to greater glucose-sensing performances. With the 50 mg L^−1^ PAA aqueous solution, the prepared PNCs showed a color change from green to red as C_g_ increased from 0 to 11 mM, with a wavelength shift from 556.3 to 625.9 nm. This change was visible to the naked eye, making the PNCs prepared under these conditions convenient for glucose analysis. Figure 7c depicts the glucose-sensing performances of PNCs prepared at different C_PAA_, with a feed molar ratio of HEAAm to AFPBA of 4.5. The changes in the initial diffraction peak and wavelength shift of the PNCs are similar to those in Figure 7b. However, the PNCs prepared at the same C_PAA_ exhibited a larger initial diffraction peak and a shorter wavelength shift. This was mainly due to the increased HEAAm feeding, leading to better initial hydrophilicity of the prepared PNCs, while the proportion of AFPBA in the hydrogel slightly decreased, resulting in a decrease in the maximum wavelength shift.

**Figure 8:**
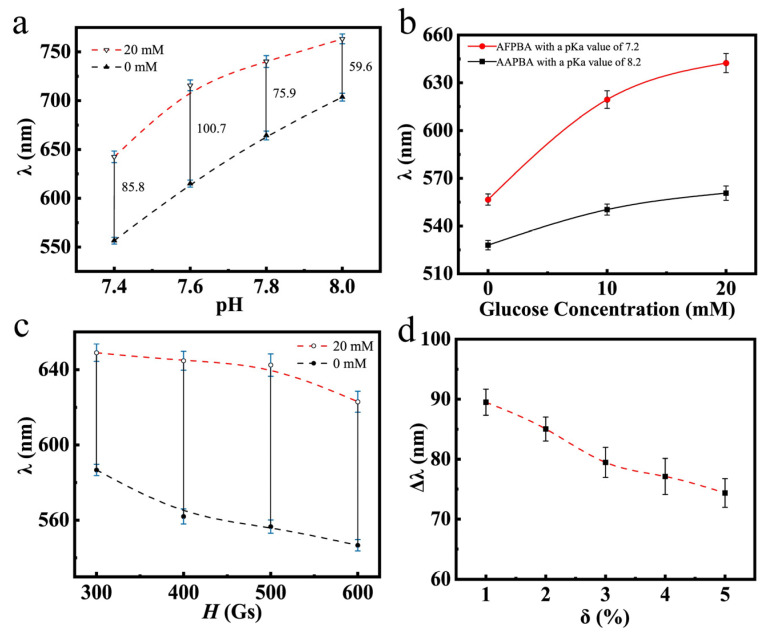
The diffraction wavelength of Fe_3_O_4_@(PVP-PAA)@poly(AFPBA-co-HEAAm) PNCs with different C_g_. (**a**) In PBS buffer at different pH values; (**b**) with different glucose-sensing monomers, (**c**) varying the magnetic field strength (*H*) and (**d**) the amount of the added crosslinking agent (*δ*) in the polymerization.

Figure 8a illustrates the glucose-sensing performances of typical PNCs in PBS buffer at different pH values. It can be observed that as the pH increased, the wavelength shift first increased and then decreased. This was mainly because at the pH values of 7.8 and 8.0, the dissociation degree of AFPBA reached 80% and 86% (Table S1), respectively, with the majority of borate anions in solution. At these pH values, the PNCs are hydrophilic enough, and the hydrophobic–hydrophilic transition of the few molecules in uncharged form had a little impact on the hydrophilicity of the polymer segments, resulting in a weakening response to C_g_. In Figure 8b, the glucose-sensing performances of the PNCs using different glucose-sensing monomers are shown. The glucose-sensing performance of PNCs obtained using AFPBA as the monomer was significantly better than that of PNCs obtained using AAPBA. This was mainly due to the much lower pKa value of AFPBA (7.2) compared to AAPBA (8.2). This difference allowed AFPBA to produce more borate anions (Table S1) to bind glucose at the pH of 7.4. On the other hand, the higher pKa value of AAPBA made it difficult to ionize and produce borate anions at the pH of 7.4, resulting in a poor glucose-sensing performance. Figure 8c,d show the relationship between the diffraction wavelength of the PNCs and different preparation conditions obtained by varying magnetic field (*H*) and crosslinker usage (*δ*). In Figure 8c, as *H* increased, the prepared PNCs exhibited a blue shift in a PBS buffer solution without glucose. It is easy to understand that a strong magnetic field compressed the space between the nanoparticles during polymerization, leading to the diffraction of shorter wavelengths. The optimal magnetic field chosen was 500 Gs, as it provided the maximum response to glucose. At 300 Gs, the distance between the nanoparticles was large, resulting in a yellow initial diffraction peak, which could not be detected visually. Therefore, the ideal *H* selected was 500 Gs. An increase in *δ* led to an increase in crosslinking density, thereby improving the rigidity of the PNCs. As a result, the expansion and contraction ability of the hydrogel was limited, leading to a decline in the wavelength shift when C_g_ changed from 0 mM to 20 mM (Figure 8d). It is also evident that the initial diffraction peak and response range of the PNCs can be adjusted to some extent by varying *H* and *δ* during polymerization.

### 3.5. Application of Fe_3_O_4_@(PVP-PAA)@poly(AFPBA-co-HEAAm) PNCs to Detect C_g_ in Artificial Serum 

**Figure 9:**
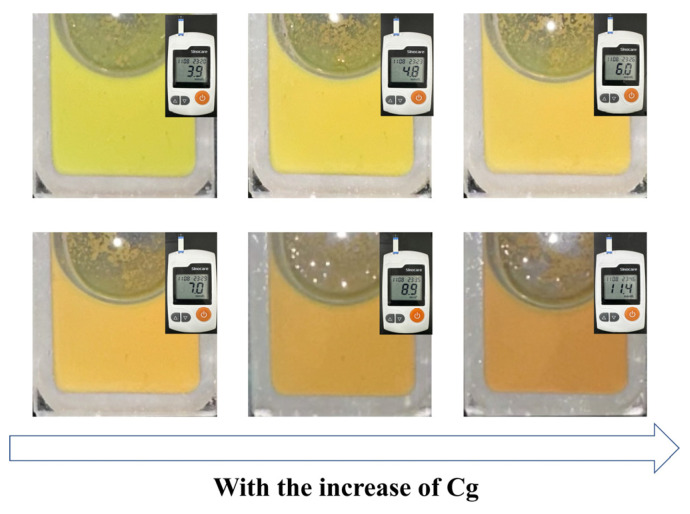
The color changes in Fe_3_O_4_@(PVP-PAA)@poly(AFPBA-co-HEAAm) PNCs in artificial serum with the increase in C_g_.

In order to verify the glucose-sensing performance of the Fe_3_O_4_@(PVP-PAA)@poly(AFPBA-co-HEAAm) PNCs in a real physiological environment, we dispersed the PNCs in artificial serum which contained glucose, inorganic salts and various proteins. C_g_ was measured by a blood glucose meter. As shown in Figure 9, the PNCs displayed a yellow-green color when they were firstly dispersed in the artificial serum at the C_g_ of 3.9 mM. As C_g_ gradually increased, the color of the PNCs continuously shifted towards the red. It can be observed that the diffraction color of the PNCs changed from yellow to orange when C_g_ increased from 6 mM to 7 mM. It was proved that the C_g_ of 7 mM is critical for diabetes detection [33]. The diffraction color of the PNCs changed from orange to red when C_g_ increased from 9 mM to 11 mM, with 11 mM also being a critical concentration for diabetes detection [33]. The CMYK values of different colors are displayed in Figure S5. The Fe_3_O_4_@(PVP-PAA)@poly(AFPBA-co-HEAAm) PNCs showed good resistance to salt and large molecular proteins, as well as maintained a high sensitivity to glucose. In general, the diffraction color of the PNCs changed from yellow-green to red as C_g_ increased from 3.9 mM to 11.4 mM, providing good naked-eye visibility and showing great potential for applications in real physiological environments within the human body.

## 4. Conclusions

In summary, we described for the first time a new type of glucose-sensing PNCs that exhibited high sensitivity in the colorimetric sensing of artificial serum as well as a rapid response within seconds. The two most important elements in constructing the PNCs are PAA and AFPBA. PAA enhanced the hydrophilicity of the PNCs by absorbing HEAAM on the Fe_3_O_4_@PVP particles via hydrogen bonding before polymerization. AFPBA, with a pKa value of 7.2, facilitated the dissociation of hydrophilic borate anions that bind glucose molecules in simulated physiological conditions. As a result, the diffraction peak of the PNCs shifted continuously and reversibly from 556.3 nm to 641.4 nm as Cg changed from 0 to 20 mM in PBS buffer at the pH of 7.4. Furthermore, a color change in the PNCs from yellow-green to red was also demonstrated in artificial serum. The continuous and fast response capabilities of PNCs along with their color-changing ability in artificial serum make them promising for applications in the field of naked-eye CGM. The PAA-assisted synthesis method will also offer help to achieve the detection of other molecules with quick responsiveness.

## Data Availability

The data presented in this study are available on request from the corresponding author.

## Supplementary Materials

The following supporting information can be downloaded at: https://www.mdpi.com/article/10.3390/nano14110964/s1, Figure S1: SEM image of the Fe_3_O_4_@PVP CNCs; Figure S2: Wavelength shift of the glucose-sensing PNCs in PBS buffer with different saccharides; Figure S3: TG curves of Fe_3_O_4_@(PVP-PAA)@poly(AFPBA-co-HEAAm) PNCs prepared under different C_PAA_ with a feed molar ratio of HEAAm to AFPBA of 4.0. (a) No addition of PAA; (b) 100 mg L^−1^ PAA aqueous solution; (c) 200 mg L^−1^ PAA aqueous solution; Figure S4: The hydrogen bond energies of HEAAm with DMSO and H_2_O calculated using Gaussian09 and Multiwfn; Figure S5: The CMYK values of the PNCs in artificial serum with the increase in Cg; Table S1: The degree of dissociation of AAPBA and AFPBA at different pH values; Table S2: The content of Fe and B by ICP analysis in Fe_3_O_4_@(PVP-PAA)@poly(AFPBA-co-HEAAm) PNCs prepared under different C_PAA_ with a feed molar ratio of HEAAm to AFPBA of 4.0; Table S3: The content of each component in Fe_3_O_4_@PVP@poly(AFPBA-co-HEAAm) PNCs prepared under different C_PAA_ with a feed molar ratio of HEAAm to AFPBA of 4.0.
