# Supplementary material for: Photonic Nanochains for Continuous Glucose Monitoring in Physiological Environment
Source: Nanomaterials (Basel). 2024 Jun 1;14(11):964. doi: 10.3390/nano14110964 (PMC11174108; doi:10.3390/nano14110964)
Supplement: Supplementary file 1 [file nanomaterials-14-00964-s001.zip › nanomaterials-3006913-supplementary.pdf]

Supplementary Information for Nanomaterials.

Photonic Nanochains for Continuous Glucose Monitoring in Physiological Environment

Gongpu Shi [1], Luying Si [1], Jinyang Cai [2], Hao Jiang [1], Yun Liu [1], Wei Luo [2,*], Huiru Ma [3,*] and Jianguo Guan [1,4]

[1] State Key Laboratory of Advanced Technology for Materials Synthesis and Processing, International School of Materials Science and Engineering, Wuhan University of Technology, Wuhan 430070, China; 270578@whut.edu.cn (G.S.); siluying@whut.edu.cn (L.S.); jh1998@whut.edu.cn (H.J.); liuyun650403@whut.edu.cn (Y.L.); guanjg@whut.edu.cn (J.G.)
[2] School of Materials Science and Engineering, Wuhan University of Technology, Wuhan 430070, China; caijinyang@whut.edu.cn
[3] School of Chemistry, Chemical Engineering and Life Science, Wuhan University of Technology, Wuhan 430070, China
[4] Wuhan Institute of Photochemistry and Technology, 7 North Bingang Road, Wuhan 430083, China
* Correspondence: rowell@whut.edu.cn (W.L.); mahr@whut.edu.cn (H.M.)

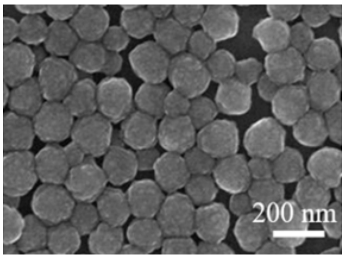

Figure S1. SEM image of the Fe_3O_4@PVP CNCs

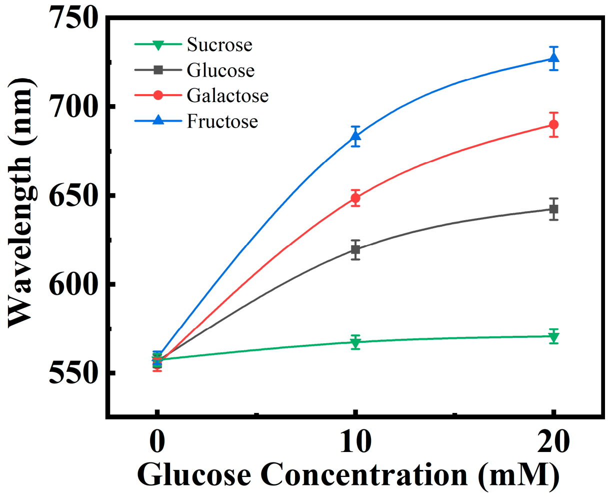

Figure S2. Wavelength-shift of the glucose-sensing PNCs in PBS buffer with different saccharides

Table S1. The degree of dissociation of AAPBA and AFPBA at different pH.

Monomer	Project	Value			
AAPBA	pH	7.4	7.6	7.8	8.0
	Degree of dissociation (%)	14	20	28	39
AFPBA	pH	7.4	7.6	7.8	8.0
	Degree of dissociation (%)	61	72	80	86

Table S2. The content of Fe and B by ICP analysis in Fe_3O_4@(PVP-PAA)@poly(AFPBA-co-HEAAm) PNCs prepared under different C_{PAA} with a feed molar ratio of HEAAm to AFPBA of 4.0.

Project	Value			
PAA concentration (mg L^{-1})	0	50	100	200
Boron content (wt%)	0.22	0.23	0.23	0.23
Fe content (wt%)	35.62	35.19	35.01	35.48

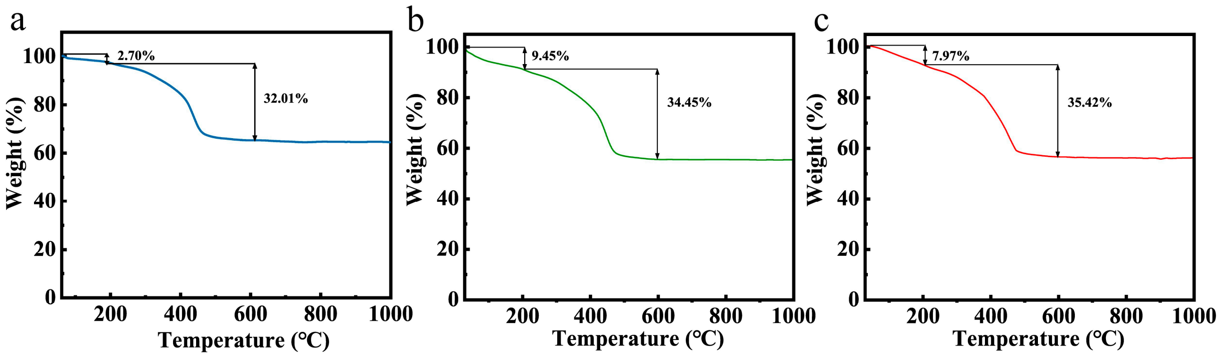

Figure S3. TG curves of Fe_3O_4@(PVP-PAA)@poly(AFPBA-co-HEAAm) PNCs prepared under different C_{PAA} with a feed molar ratio of HEAAm to AFPBA of 4.0. (a) No addition of PAA; (b) 100 mg L^{-1} PAA aqueous solution; (c) 200 mg L^{-1} PAA aqueous solution.

Table S3. The content of each component in Fe_3O_4@PVP@poly(AFPBA-co-HEAAm) PNCs prepared under different C_{PAA} with a feed molar ratio of HEAAm to AFPBA of 4.0.

Project	Value			
PAA concentration (mg L^{-1})	0	50	100	200
Fe_3O_4 content (wt%)	67.1	62.8	62.0	61.7
PVP content (wt%)	17.5	16.4	16.2	16.1
AFPBA content (wt%)	7.8	7.7	7.6	7.5
HEAAm content (wt%)	7.6	13.1	14.2	14.7
n_{HEAAm}: n_{AFPBA}	2.4	4.1	4.5	4.8

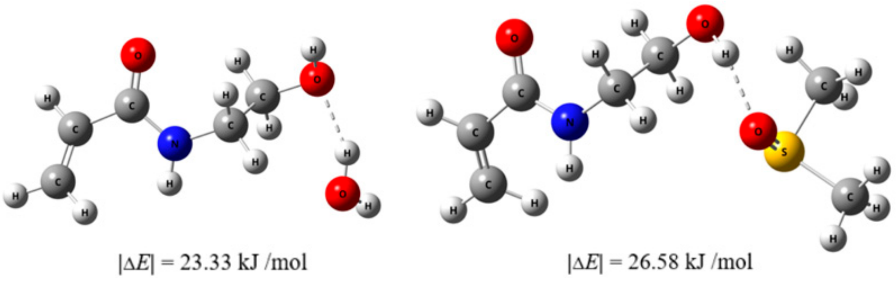

Figure S4. The hydrogen bond energies of HEAAm with DMSO and H_2O calculated using Gaussian09 and Multiwfn.

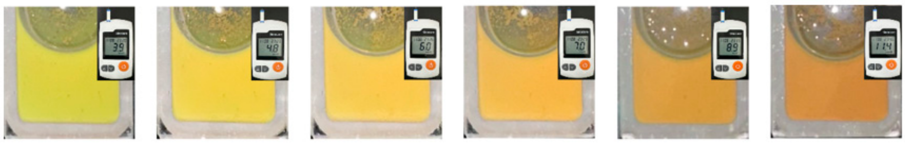

CMYK values (22,16,77,0) (14,19,76,0) (12,25,72,0) (13,33,77,0) (26,44,78,0) (30,50,78,0)

Figure S5. The CMYK values of PNCs in artificial serum with the increase of Cg
